# Metalloproteinases and their inhibitors are influenced by inhalative glucocorticoid therapy in combination with environmental dust reduction in equine recurrent airway obstruction

**DOI:** 10.1186/s12917-016-0915-1

**Published:** 2016-12-09

**Authors:** Ann Kristin Barton, Tarek Shety, Angelika Bondzio, Ralf Einspanier, Heidrun Gehlen

**Affiliations:** 1 Equine Clinic, Freie Universitaet Berlin, Oertzenweg 19b, Berlin, 10163 Germany; 2 Institute of Veterinary Biochemistry, Freie Universitaet Berlin, Oertzenweg 19b, Berlin, 10163 Germany

**Keywords:** Horse, Lung, Inflammatory marker, Recurrent airway obstruction, Metalloproteinases, Tissue inhibitors of metalloproteinases

## Abstract

**Background:**

Overexpression of matrix-metalloproteinases (MMPs) has been shown to lead to tissue damage in equine recurrent airway obstruction (RAO), as a misbalance with their natural inhibitors, the tissue inhibitors of metalloproteinases (TIMPs), occurs. This favors irreversible pulmonary fibrosis formation. Increased levels of MMPs, TIMPs or altered ratios between them can be used as biomarkers of respiratory disease. We hypothesized that levels of MMPs, TIMPs and their ratios correlate with improvement in clinical findings and bronchoalveolar lavage fluid (BALF) cytology after 10 days of inhalative glucocorticoid therapy and environmental dust reduction (EDR) and may be used to monitor treatment success.

Ten horses with a history of RAO participated in a prospective clinical study. Clinical and cytological scoring was performed before and after inhalative therapy using budesonide (1500 μg BID over 10 days) and EDR (bedding of wood shavings and wet hay as roughage). Gelatin zymography was performed for qualitative and semi-quantitative evaluation of MMP-2 and MMP-9 in BALF supernatant, while fluorimetry was used to evaluate MMP-8 activity. Additionally, specific equine ELISA assays were used for quantitative assessment of MMP-2, MMP-9, TIMP-1 and TIMP-2.

**Results:**

A significant reduction in the total and several single parameters of the clinical score were found after 10 days of inhalative therapy and EDR. The concentrations of MMP-2, MMP-9, TIMP-1 and TIMP-2 (ELISA) as well as their activities (MMP-2 and MMP-9 zymography and MMP-8 fluorimetry) were significantly decreased after therapy. Significant improvements in MMP-8/TIMP-1 and MMP-8/TIMP-2 ratios were also found, differences between other ratios before and after therapy were insignificant.

**Conclusions:**

Metalloproteinases and their inhibitors, in particular MMP-9 and TIMP-2, are valuable markers for clinical improvement in RAO.

## Background

Severe equine asthma, commonly known as recurrent airway obstruction (RAO), is a very common pneumopathy in middle-aged to older horses with a prevalence of about 14% in the general horse population [1]. Older studies even found an incidence over 50% of this respiratory disease [2]. Thus, RAO is considered an important problem requiring veterinary attention as it leads to decreased performance capacity and major economic losses [3].

Equine RAO resembles human asthma and COPD in many ways. In all of them, so called remodeling of the pulmonary tissue occurs, which includes reduction of bronchial luminal caliber, smooth muscle hypertrophy, peribronchiolar fibrosis formation and airway epithelial cell hyperplasia, all impeding gas exchange [4, 5]. Control of remodeling may be the key to effective therapy and long-term success of managing these patients [6].

The extracellular matrix (ECM) stabilizes the inner structures of the lung, for which collagen is the most important ECM component. Therefore, the ECM is essential to insure the efficacy of gas exchange. To allow for growth and physiologic tissue repair, it underlies a continuous turnover, but a physiologic balance between synthesis and degradation needs to be maintained. Cleavage of ECM components takes place by zinc-depentant endopeptidases, so called matrix-metalloproteinases (MMPs) and is controlled by their natural inhibitors, the tissue inhibitors of matrix metalloproteinases (TIMPs) [6, 7]. Overwhelming proteolytic degradation in consequence of a dysbalance of MMP and TIMP activity leads to an inflammatory response and ultimately fibrosis formation [8, 9].

Several studies have been published focusing on MMP levels in equine RAO. Increased collagenolytic activity, as indicated by MMP-8 concentrations, was found in tracheal epithelium lining fluid (TELF) [10]. Levels of MMP-8 and MMP-13 were positively correlated with type-I collagen degradation [11].

Markedly increased elastinolytic activity in TELF, indicated by MMP-2 and −9, was also found in RAO [12, 13], but other authors found no disease-associated increase of MMP-2 levels and concluded that its major role may be the physiologic ECM turnover [14]. MMP-9 on the other hand shows very low levels in health, but increases dramatically during exacerbations of asthma and RAO as well as in idiopathic pulmonary fibrosis [15].

While levels of MMPs have been studied intensively in RAO, not much is known about their natural inhibitors, in particular TIMP-1 and TIMP-2, as well as the course of MMPs and TIMPs under different forms of therapy. The inhibitory effect of different tetracyclines, flunixin meglumine and pentoxifylline on elastinolytic activity after intravenous infusion of lipopolysaccharide (LPS) was evaluated [16]. All MMP inhibitors significantly decreased MMP activities, but pentoxifylline and oxytetracycline appeared to be the most effective. Collagenase activity also could be reduced by doxycycline inhibition in tracheal aspirates [10]. These authors concluded that MMP inhibitors might be a valuable new treatment approach to equine RAO. A similar effect was found in a clinical trial in human medicine, in which COPD patients with stable symptoms showed significant improvement in lung function after 4-weeks of doxycycline treatment [17].

Before testing MMP inhibitors in a clinical setting, we were interested in the course of MMPs and TIMPs under the cornerstone of RAO therapy, namely environmental dust reduction in combination with anti-inflammatory glucocorticoids, which were given in an inhalative approach to achieve high local concentrations in the lung. The focus of this study was the evaluation of possible correlations between clinical and cytological parameters with MMP-2, −8 and −9 as well as TIMP1- and −2 activities and concentrations before and after 10 days of inhalative budesonide therapy and environmental dust reduction (EDR).

## Methods

### Preparticipation examination

10 horses (4 geldings, 6 mares, age 16.5 ± 4.3 years, BDW 481 ± 80.4 kg) with a history of RAO were examined to evaluate the current disease status using a validated clinical score system, recommended by an international workshop [18, 19] and modified by including cytology of BALF instead of tracheal aspirates [20], the previous diagnosis was confirmed (Table 1). Horses in remission, not meeting the inclusion criteria for RAO exacerbation, but still showing parameters above reference values, were included into the study, as these patients account for a large part of our hospital population and often show further improvement under therapy.

**Table 1:** Clinical scoring system, modified from Ohnesorge et al. (1998): The highest score given in each subcategory counts as maximum points for this subcategory, maximum points of subcategories are summed up to gain the total score number

		Score	Max. Points
1. Coughing	No cough after manual compression of larynx	0	1
	from history, spontaneously or induced	1	
2. Dyspnoea at rest	moderately increased abdominal effort	1	3
	Nostril flare	3	
	Hypertrophy of abdominal muscles	3	
3. Percussion lung field	>1 hand increase	1	2
	>2 hands increase	2	
4. Lung auscultation	Wheezes and crackles	2	2
5. Endoscopy	Significantly increased secretions with moderate viscosity	1	2
	Highly increased secretions with high viscosity	2	
	Marked thickening of tracheal bifurcation [52]	1	
6. BALF cytology	Neutrophils <8%	0	3
	Neutrophils 8–15%	1	
	Neutrophils 15–25%	2	
	Neutrophils >25%	3	
7. Blood gas analysis	PAO_2_-PaO_2_: 0–7 mmHg	0	2
	PAO_2_-PaO_2_: 7–14 mmHg	1	
	PAO_2_-PaO_2_: >14 mmHg	2	

### BALF collection and processing

Under local anesthesia using 20 ml of 2% lidocaine^1^ applied to the main bronchi, bronchoalveolar lavage was performed using a silicone catheter.^2^ Five hundred milliliters of pre-warmed phosphate buffered saline^3^ were infused and immediately aspirated. BALF was divided into 2 aliquots for cytological and biochemical examination. After centrifugation^4^ (250 g, 10 min at 4 °C) the BALF supernatant was kept at −80 °C until further analysis. Cytology was performed using Wright-Giemsa staining and counting 500 cells at 500x magnification.

### Gelatine zymography of elastases

Zymography was performed^5^^,^ 6 following the manufacturer’s instructions. Human MMP-2 and MMP-9 controls were used together with a multicolor broad protein range protein ladder as a control on each gel. Also, a sample of a healthy control horse (samples of a previous study) was applied to each gel to compare the signals to affected horses. Gels were scanned for digital analysis by densitometry using digital image analyzing software^7^ to quantify the bands objectively as described previously [21].

### ELISA of MMPs and TIMPs

Commercially available ELISA systems were used for quantification of MMP-2, MMP-9, TIMP-1 and TIMP-2 concentrations.^8^ ^9^ ^10^ ^11^ Duplicate standards and samples were measured following the manufacturer’s instructions and absorbance measured with an ELISA microplate reader^12^ at 450 nm.

### Fluorimetry MMP-8 assay

For the MMP-8 assay^13^ the manufacturer’s instructions were followed. Negative controls containing assay buffer and positive controls using recombinant human purified MMP-8 were included.

### Inhalation therapy

Inhalation therapy was performed twice daily at a dosage of 1500 μg budesonide (Pulmicort™ suspension)^14^ using an automatic inhalation device^15^. Although the study participants had been under environmental dust reduction (wood shavings as bedding and wet hay as roughage) at their home stables prior to the study, they were stabled in the clinic for overall 16 days to ensure the same conditions concerning stabling and application of inhalation therapy for all animals. During the phase of inhalation therapy a daily clinical examination ensured that no horse was suffering from complications or showed clinical signs of respiratory infection.

### Check-up examination

After 10 days of inhalation therapy, the horses were examined following the same protocol as for the preparticipation examination exclusive of thoracic radiography. Afterwards, they were discharged from the clinic with instructions to the owners concerning further management and therapy.

### Statistical analysis

Statistical analysis was performed using SPSS Statistics^16^ and results were expressed as mean ± standard deviation (SD). As parts of data were not found to be normally distributed using the Shapiro Wilks Test, non-parametric tests were used for the entire analysis. The level of significance was set at *P* < 0.05.

Spearman rank correlation coefficients were calculated between clinical and cytological parameters, MMP and TIMP concentrations or activities, respectively. The level of significance was set at *P <* 0.05.

The Wilcoxon signed ranks test was used to analyze differences before and after budesonide inhalation in combination with environmental dust reduction Again, the level of significance was set at *P <* 0.05.

## Results

### Clinical scoring

The anamnestic diagnosis of RAO was confirmed for all 10 horses. 7 horses were classified RAO in exacerbation, 3 in partial remission. All were subjected to treatment. The total examination score before and after therapy showed a significant difference before and after therapy (*P =* 0.005) with the endoscopy score being the single parameter of highest significance (*P =* 0.007).

Inhalation therapy and environmental dust reduction resulted in significantly decreased neutrophil ratio after therapy (25.7 ± 19.4%) compared to before therapy (42.92 ± 27%). As neutrophils decreased (*P =* 0.013), the relative percentage of macrophages increased (*P =* 0.022) from 32.77 ± 18.7% before therapy to 45 ± 16.57% after therapy.

Results of clinical scoring are shown in Table 2.

**Table 2:** Mean results ± standard deviation (Min-Max) of clinical scoring before and after therapy in 10 RAO horses

Parameter	Before therapy	After therapy	*P*
Coughing	0.5 ± 0.53 (0–1)	0.3 ± 0.48 (0–1)	0.157
Dyspnea at rest	1.1 ± 1.1 (0–3)	0.7 ± 0.82 (0–2)	0.046*
Percussion of the lung field	0 ± 0 (0–0)	0 ± 0 (0–0)	1
Lung auscultation	0 ± 0 (0–0)	0 ± 0 (0–0)	1
Endoscopy	1.5 ± 0.53 (1–2)	0.6 ± 0.52 (0–1)	0.007*
BALF cytology	2.4 ± 1.1 (0–3)	2 ± 1.25 (0–3)	0.157
Blood gas analysis	0.4 ± 0.7 (0–2)	0 ± 0 (0–0)	0.102
Total clinical score	6 ± 2.6 (2–10)	3.1 ± 2^*↓^ (0–5)	0.005*

* marks significance at *P <* 0.05

### MMP-2 ELISA

The concentration of MMP-2 was significantly decreased after therapy (3.54 pg/ml ± 0.71 pg/ml) compared to before therapy (4.93 pg/ml ± 0.91 pg/ml, *P =* 0.005). MMP-2 activity in BALF detected by gelatin zymography was significantly decreased after therapy (5827.45 ± 5250.42) compared to that before therapy (12570.98 ± 9164.32, *P =* 0.028).

### MMP-9 ELISA

MMP-9 concentration in BAL was significantly decreased after therapy (282.29 ng/ml ± 101.19 ng/ml) compared to before therapy (414.59 ng/ml ± 119.6 ng/ml, *P =* 0.005). The MMP-9 activity in gelatin zymography was significantly decreased after therapy (2942.95 ± 2440.24) compared to before therapy (11176.68 ± 11255.3, *P =* 0.022).

**Fig. 1:**
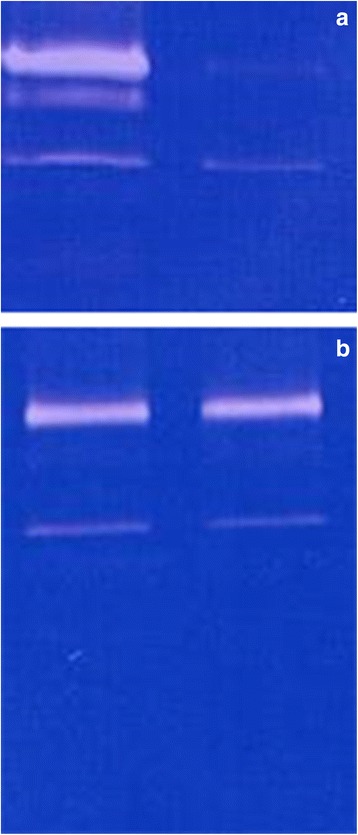
Zymography of MMP-2 (*lower band*) and MMP-9 (*upper band*) before (*left*) and after (*right*) inhalation therapy in a horse showing clear improvement in clinical and cytologic data (**a**) versus a horse, in which therapy did not lead to significant improvement (**b**)

Examples of MMP-2 and MMP-9 zymography before and after therapy are shown in Figs. 1a and b.

### TIMP-1 ELISA

TIMP-1 concentration was significantly decreased after therapy (249.78 ± 59.56 pg/ml) compared to before therapy (308.80 ± 7.44 pg/ml, *P =* 0.005).

### TIMP-2 ELISA

TIMP-2 concentration was also significantly decreased after therapy (18.76 ± 2.79 ng/ml) compared to before therapy (26.20 ± 1.49 ng/ml, *P =* 0.002).

### MMP-8 fluorimetry

The activity of MMP-8 was significantly decreased after therapy (0.10 RFU ± 0.11 RFU) compared to before therapy (0.57 RFU ± 0.85 RFU, *P =* 0.005).

Results of MMP- and TIMP-measurements are presented in Table 3.

**Table 3:** MMP-2, MMP-9, TIMP-1 and TIMP-2 ELISA and MMP-8 fluorimetry measurements, the results are expressed as mean ± SD

	n	Before therapy	After therapy	*P*-value
MMP-2 ELISA [pg/ml]	10	4.93 ± 0.91	3.54 ± 0.71	0.005
MMP-9 ELISA [ng/ml]	10	414.59 ± 119.6	282.29 ± 101.19	0.005
TIMP-1 ELISA [pg/ml]	10	308.80 ± 7.44	249.78 ± 59.56	0.005
TIMP-2 ELISA [ng/ml]	10	26.20 ± 1.49	18.76 ± 2.79	0.002
MMP-8 Fluorimetry [RFU]	10	0.57 ± 0.85	0.1 ± 0.11	0.005
MMP-2 zymography	10	12570.98 ± 9164.32	5827.45 ± 5250.42	0.028
MMP-9 zymography	10	11176.68 ± 11255.3	2942.95 ± 2440.24	0.022

### Correlation of clinical score and MMPs/TIMPs

Strong positive correlations were found between the total clinical scoring and concentrations of MMP-2 (*r =* 0.66), MMP-9 (*r =* 0.83), TIMP-1 (*r =* 0.65), TIMP-2 (*r =* 0.72) concentrations, MMP-8 activity (*r =* 0.75) and weakly for MMP-2 (*r =* 0.47) activity measured by gelatin zymography. All these correlations had a level of significance of *P <* 0.01, except MMP-2 zymography (*P =* 0.036). No significant correlation was found between the clinical examination score and MMP-9 zymography (*r =* 0.30).

### Correlations of BALF cytology and MMPs/TIMPs

BAL neutrophil percentages showed no correlation with the concentrations of MMP-2 (*r =* 0.39), but significant correlations with MMP-9 (*r =* 0.59), TIMP-1 (*r =* 0.65), TIMP-2 (*r =* 0.72) concentrations and MMP-8 activity (*r =* 0.78) with *P <* 0.01. MMP-2 activity measured by gelatin zymography was also weakly correlated (*r =* 0.45), while MMP-9 (*r =* 0.25) activity was not (*P <* 0.05).

### MMP/TIMP ratios

Significant differences were found for MMP-8 / TIMP-1 (*P =* 0.002) and MMP-8 / TIMP-2 (*P =* 0.038) ratios before and after therapy as shown in Table 4.

**Table 4:** MMP/TIMP ratios before and after therapy (*n =* 10)

	MMP-2/ TIMP-1	MMP-2/ TIMP 2	MMP-9/ TIMP-1	MMP-9/ TIMP-2	MMP-8/ TIMP-1	MMP-8/ TIMP-2
Before therapy	0.016	0.188	1.343	15.824	0.002	0.022
After therapy	0.014	0.188	1.130	15.047	0.000*	0.005*

The (*) shows significance at *P <* 0.05

## Discussion

Pathogenesis of airway remodeling – In human asthma, activation of fibroblasts leads to increases of ECM mass by overwhelming fibrosis formation, a process similar to bronchial remodeling in equine RAO. This is suspected to be the consequence of a pathologic dysbalance between proteolytic degradation of ECM components by MMPs, and excessive tissue repair primarily mediated by TIMP-1, which can be upregulated very effectively and is capable of cleaving all active MMPs. Elastinolytic and collagenolytic activity was found in several studies on human asthma, in particular increased levels of MMP-9 [22, 23], but also MMP-2 [24, 25]. Former studies of our group showed that this is also true for several equine pneumopathies including RAO, inflammatory airway disease (IAD) and chronic interstitial pneumopathy compared to healthy controls [26, 27]. Compared to these controls, all horses of the presented study showed increased levels of MMP 2, 8 and 9 prior to therapy. Increased TIMP levels have also been found in humans suffering from asthma [23, 24, 28, 29] and the same was found for equine RAO in this study and previous [26, 27]. This may allow the suspicion that fibrosis formation as a long-term consequence of human asthma and equine RAO may be the ultimate result of continuous activation of tissue repair mechanisms or over-repair processes [30]. The most important source of MMP-8, MMP-9 and TIMP-1 are neutrophils, which are typically increased in RAO, accounting for more than 25% of BALF cytology by definition [1]. Therefore, decreases of these enzymes in accordance with decreasing percentages of neutrophils can be expected.

Effects of corticosteroids on MMPs and TIMPs - According to the GINA (global initiative for asthma) guidelines, inhalative therapy using various corticosteroids including budesonide is the key factor of asthma therapy after the control of acute disease exacerbation and is commonly used for persistent long-term management [31]. Reduced pulmonary function and increased inflammatory mediators are also found in equine RAO during phases of clinical remission [32] and corticosteroid treatment in combination with ß_2_-mimetic bronchodilatators and secretolytic therapy is accepted as the most successful therapy after consequent environmental dust-control [1]. Inhalative treatment is becoming more and more popular in equine medicine and results may be more comparable to what is known in asthmatic patients, so we chose this route of application for our study. Glucocorticoids were shown to slow down the process of bronchial remodeling by downregulation of elastinolytic activity and TIMP upregulation at the same time [33], but other authors could not confirm this observation. Data of the presented study shows indeed the most significant decrease in MMP-9 compared to MMP-2 and 8, but there were also significant decreases in TIMP-1 and TIMP-2. Ex vivo studies demonstrated the ability of corticosteroids to decrease MMP-9 and TIMP-1 levels [34]. In human lung fibrosis, MMP-9 expression could be inhibited with steroids and immunosuppressants [35]. Fibrosis formation is also a long-term effect of equine RAO [36, 37] and it might be suspected that TIMPs decrease in reaction to decreasing MMPs. Nevertheless, ratios of elastases and TIMPs did not differ before and after inhalative therapy in combination with environmental dust control in the presented study.

Todorova et al. [30] studied the effect of inhaled budesonide combined with the bronchodilator formoterol on the metalloproteolytic balance between MMPs and TIMPs. This combination therapy inhibited upregulation of prostaglandine and TIMP-1 production, MMP-9 levels and the MMP-9 / TIMP-1 ratio in human lung fibroblasts, whereas MMP-2 was not affected. Budesonide monotherapy required higher concentrations and formoterol alone had no effects. Therefore, the synergistic effect of budesonide and formoterol was considered the best option to reduce enhanced metalloproteolytic activity and may inhibit or at least reduce pulmonary fibrosis formation in asthma. We might have enhanced the effect of our therapy on MMPs in horses, if the budesonide had been combined with a ß_2_-agonist like clenbuterol, which is commonly used in horses. This should be the subject of further studies. In asthmatic rats sensitized by ovalbumin and challenged with mist inhalation, steroid treatment not only reduced MMP-9 and TIMP-1 levels, but also improved hyperplasia of airway smooth muscle and basement membrane [38]. Inhalation of budesonide only influences MMP-9 and TIMP-1 levels in moderate to severe disease though, no effect of inhaled budesonide was found in subjects suffering from mild asthma [25].

Obase et al. [39] studied the effects of inhaled budesonide in children with mild and moderate symptoms of asthma on MMP-8 and TIMP-1 levels in induced sputum. Before the beginning of inhalation, MMP-8 was higher in asthma of moderate severity, TIMP-1 was lower and the MMP-8/TIMP-1 ratio was higher in mild and moderate asthma in comparison to healthy childeren. TIMP-1 levels increased in consequence of budesonide inhalation leading to normalization of the the MMP-8 / TIMP-1 ratio. Nevertheless, the excessive MMP-8 levels remained in the airways of children with moderate symptoms. Therefore, corticosteroids may be capable of controlling asthma symptoms, but may be insufficient in preventing the occurrence of airway remodeling involving MMP-8. Results of the presented study, however, show a significant reduction in MMP-8 activity after corticosteroid therapy, although most horses were in disease exacerbation before starting the inhalation protocol. There were also significant reductions in both TIMP concentrations as well as in the MMP-8 / TIMP-1 and the MMP-8 / TIMP-2 ratios. This shows that the MMP-8 / TIMP ratios in the horse do not normalize by upregulation of TIMP, but by downregulation of MMP-8. This seems pleasing and would improve prognosis in equine disease compared to human asthma, but further studies should be performed on the role of MMP-8, as airway remodeling is definitely a long-term effect of equine RAO leading to irreversible exercise insufficiency, but fibrosis formation might not be a direct consequence of TIMP overexpression in the horse.

New therapeutic approaches - MMPs themselves have been discussed as an ideal target for new therapeutic approaches as they play an active role in disease pathophysiology [40]. Inhibition of MMP activity has been studied in human cancer. Although this was not successful [41], new data suggests that selective inhibitors might find a role in acute and chronic anti-inflammatory therapy after all [42]. This includes asthma, where reduced airway inflammation and hyperresponsiveness was reported after local bronchial application of synthetic TIMP-2 [43]. TIMP-2 downregulated MMP-2 activity [44]. In addition to their antibiotic acitivity, tetracyclines act as effective MMP inhibitors. Doxycycline was studied in asthma models including different species [10, 45, 46]. After oral administration in mice, it reduced airway inflammation and hyperresponsiveness as well as MMP-9 expression. Further studies have shown that MMP-inhibition also decreases goblet cell hyperplasia, another component of airway remodeling in asthma [47], whichh is also known for equine RAO. In the horse, in-vitro studies showed reductions of proteolytic activity using acetylcysteine, pentamidine and diminazene [14, 48]. The clinical application of synthetic TIMPs in RAO horses is currently under consideration by our group.

Limitations - To our knowledge, this is the first study on the course of MMPs in RAO affected horses and a clinical trial, but we were faced with several limitations, so unintentional bias may have occurred at several points. Due to the fact that privately owned horses were included to the study, it was doubtful if stabling of all horses at their home stables was actually in a low-dust environment, as most horses were still in exacerbation and had increased MMPs at the beginning of therapy. The effect of therapy may therefore be based on the combined effect of inhalative glucocorticoid therapy and environmental dust reduction under clinic conditions, with the effect of the latter being quite variable. Even if horses had been stabled on wood shavings and fed wet hay, they may have come from a very dusty environment apart from these two conditions for enrolment in the study. A second group subjected to a low-dust-environment in the clinic only would have been preferable to control this effect and was planned to be included in the study, but unfortunately, not enough owners agreed to this protocol. Therefore, the actual bias caused by this is hard to assess. Another time-point of sampling after a few days of low-dust-environment before starting inhalation therapy would have been helpful as well, but again, owners wished for hospitalization as short as possible. On the other hand, it was not the aim of this study to prove the therapeutic effect of neither environmental dust reduction, which is commonly acceptected [1] nor inhaled budesonide, which has also already been published [49], but to study the course of MMPs and TIMPs under clinical improvement to evaluate their usefulness as biomarkers and correlation with clinical and cytological findings. Clinical scoring and even the quantitative tests may have been subject to bias as well. The clinical scoring was done by two independent observers, but these were not blinded to horses’enrolment in the study and the time-point as before or after therapy, which was due to the fact that subjects enrolled were clinic patients. Endoscopy and cytology was done by one observer and images scored by another later on randomly and blinded. ELISAs and zymography were performed, when all samples had been collected and stored, also randomly and blinded.

## Conclusion

Despite the difficulties discussed above, the results of the presented study suggest that developing new therapeutic approaches to correct the misbalance between MMPs and TIMPs might be promising for treatment of asthma and equine RAO, which shares many features with human asthma disease. Due to their various effects in different organ systems, it seems safer to target MMPs actually relevant in this disease specifically and to use an inhalative approach to reach high local levels of the drug, while the remaining organism remains unaffected. In chronic pneumopathies, targeting MMP-2 and MMP-8, but in particular MMP-9 might be of therapeutic value [50]. First attempts in this direction have been taken in treating cancer, but less data is available on inhibition of MMP activity in chronic pneumopathies [51].

To our knowledge, TIMP-1 or TIMP-2 expression in equine RAO has not been studied so far. Studies in horses on the effect of inhaled glucocorticoids on MMPs or TIMPs have not been published either. Therefore, this is the first clinical study to show a correlation of MMP and TIMP levels to clinical findings and cytology. Parallels to results presented in papers on humans and laboratory animals allow the suspicion that the same mechanisms might apply in the horse regarding the effects of glucocorticoids, but further studies including a control group have to be performed to differentiate a possible therapeutic effect between glucocorticoid inhalation and envirnonmental dust reduction in horses affected by RAO.

## Abbreviations

AaDo_2_: Arterio-alveolar pressure difference
BALF: Bronchoalveolar lavage fluid
BDW: Body weight
BGA: Blood gas analysis
BID: Bis in die, twice daily
CIP: Chronic interstitial pneumopathy
COPD: Chronic obstructive pulmonary disease
ECM: Extracellular matrix
EDR: Environmental dust reduction
ELISA: Enzyme-linked immunosorbent assay
GINA: Global initiative for asthma
IAD: Inflammatory airway disease
LaGeSo: State Office of Health and Social Affairs Berlin
LPS: Lipopolysaccharide
MMP: Matrix metalloproteinase
PaO_2_: Partial oxygen pressure
RAO: Recurrent airway obstruction
Rpm: Rounds per minute
SD: Standard deviation
TELF: Tracheal epithelium lining fluid
TIMP: Tissue inhibitor of metalloproteinases

## Footnotes

1 Lidocaine, bela-Pharm GmbH, Vechta, Germany

2 Silicone Bronchoalveolar Lavage Catheter 300 cm, Smiths Medical ASD, Inc, USA

3 Phosphate buffered saline, Lonza, Verviers, Belgium;^d^Table Top Refrigerated Centrifuge Hermle Z326K, Hermle Labortechnik GmbH, Germany

4 Gelatin zymogram gels*,* Life technologies, USA

5 electrophoresis device XCell, Novex Experimental Technology, Japan

6 ImageJ v1.47, Wayne Rasband, NIH, USA

7 equine MMP-2 kit, USCN Life Science Inc., China

8 equine MMP-2 kit, USCN Life Science Inc., China

9 equine MMP-9 kit, USCN Life Science Inc., China

10 equine TIMP-1 kit, USCN Life Science Inc., China

11 equine TIMP-2 kit, USCN Life Science Inc., China

12 ELISA microplate reader, BioRad Laboratories, USA

13 Sensolyte^TM^ 520 MMP-8 Assay Kit, Anaspec, Inc. Fermont, USA

14 Pulmicort™ suspension, Astra Zeneca, Germany

15 SaHoMa II mobile ultrasonic nebulizer, NEBU-TEC International, Germany

16 SPSS Statistics, Version 17.0 released 2008, SPSS Inc., USA
